# A comprehensive multi-layered analysis reveals genetic pleiotropy underlying coronary artery calcification and bone mineral density

**DOI:** 10.1016/j.bone.2025.117719

**Published:** 2025-11-06

**Authors:** Tao Han, Yang Qu, Jiangbo Zhu, Linna Sha, Bowen Lei, Rong Xiang, Xunying Zhao, Jiaojiao Hou, Qin Deng, Sirui Zheng, Jinyu Zhou, Ting Yu, Xin Song, Bin Yang, Yangdan Zhong, Maoyao Xia, Douglas P. Kiel, Xia Jiang

**Affiliations:** a Department of Nutrition and Food Hygiene, West China School of Public Health, West China Fourth Hospital, Sichuan University, Chengdu, Sichuan, China; b Department of Epidemiology and Biostatistics, West China-PUMC C. C. Chen Institute of Health, West China School of Public Health, West China Fourth Hospital, Sichuan University, Chengdu, Sichuan, China; c Department of Clinical Neuroscience, Center for Molecular Medicine, Karolinska Institute, Solna, Stockholm, Sweden; d Hinda and Arthur Marcus Institute for Aging Research, Hebrew Senior Life, Boston, MA, 02131, United States; e Department of Medicine, Beth Israel Deaconess Medical Center, Harvard Medical School, Boston, MA, 02215, United States

**Keywords:** Coronary artery calcification, Bone mineral density, Genetic correlation, Pleiotropic loci, Calcification paradox

## Abstract

**Objectives:**

Subclinical atherosclerosis and osteoporosis are often present together in the same individual, but their common mechanisms remain unclear. This study aims to investigate the pleiotropic relationship underlying coronary artery calcification (CAC) and estimated calcaneal bone mineral density (eBMD), providing molecular insights into their observed phenotypic associations.

**Methods:**

Genetic correlation between CAC and eBMD was estimated based on genome-wide summary statistics. Shared loci were examined at the levels of single-nucleotide polymorphism (SNP), multi-SNP, and gene expressions to provide insights into genetic pleiotropy. Sensitivity analyses using data of DXA-derived BMD at femoral neck and lumbar spine were performed to validate key findings. Pathway enrichment analyses were conducted on significant shared loci to explore potential biological mechanisms.

**Results:**

Despite an absence of a global genetic correlation, partitioning the genome into independent regions revealed five significant signals. Through subsequent multi-layered analysis, we identified 211 non-overlapping significant shared genes, including 190 from single-SNP-level, 27 from multi-SNP-level, and 3 from gene expression level, underscoring the widespread pleiotropy across CAC and eBMD. Notably, the shared signals were predominantly concentrated on chromosome 17, with *SMG6* and *PAFAH1B1* highlighted as crucial pleiotropic genes, and both were further confirmed by sensitivity analyses. Pathway enrichment analyses revealed oxidative stress regulation and the ubiquitin-proteasome system as critical biological mechanisms potentially linking the two traits.

**Conclusion:**

Our study demonstrates that the observed association between CAC and eBMD is mainly driven by pleiotropic associations.

## 1. Introduction

The “calcification paradox” describes the inverse association between calcium content in bone and simultaneous arterial calcification [1]. This phenomenon, reflecting an unusual distribution of mineralization within the body, is often observed in clinical practice [2]. In many cases, a diagnosis of osteoporosis is associated with an increased risk of subclinical atherosclerosis and vice versa, suggesting an intricate interplay between the skeletal and the vascular systems [3,4]. Therefore, understanding the potential biological mechanisms underlying this paradox remains essential for the effective management of both osteoporosis and cardiovascular diseases.

While the exact mechanisms remain incompletely understood, accumulating evidence implies a potential shared genetic architecture. Arterial calcification has been recognized as an actively regulated process akin to ossification, rather than a passive deposition of calcium, implying a shared regulatory network promoting calcification across different systems [5]. Indeed, both carotid adventitial diameter and bone mineral density (BMD) are highly heritable traits and show significant genetic correlations [6]. Moreover, several key genetic regulators of bone formation, such as *RUNX2* and *BMP2*, are also involved in the progression of vascular calcification [7]. Similarly, critical signaling systems that govern bone remodeling, like the OPG-RANK-RANKL pathway, also play a role in vascular homeostasis [5]. Collectively, these findings indicate that a shared genetic architecture may underlie the bone-vascular interplay, yet the full extent remains insufficiently characterized.

From a genetic perspective, the relationship between traits can arise through vertical causality (where one trait causally influences the other) or horizontal pleiotropy (where genetic variants independently influence both traits). Mendelian randomization (MR) analysis is a widely used approach to detect a vertical causal relationship. However, a recent large-scale MR study found no evidence of a causal association between coronary artery calcification (CAC) and BMD measured by dual-energy X-ray absorptiometry (DXA) at several skeletal sites [8]. This crucial finding suggests that the paradox may be attributable to pleiotropy, where genetic factors influence both skeletal and vascular traits through independent pathways. To date, no study has systematically investigated the co-regulated genes and pathways pertaining to such a pleiotropy.

**Fig. 1.**
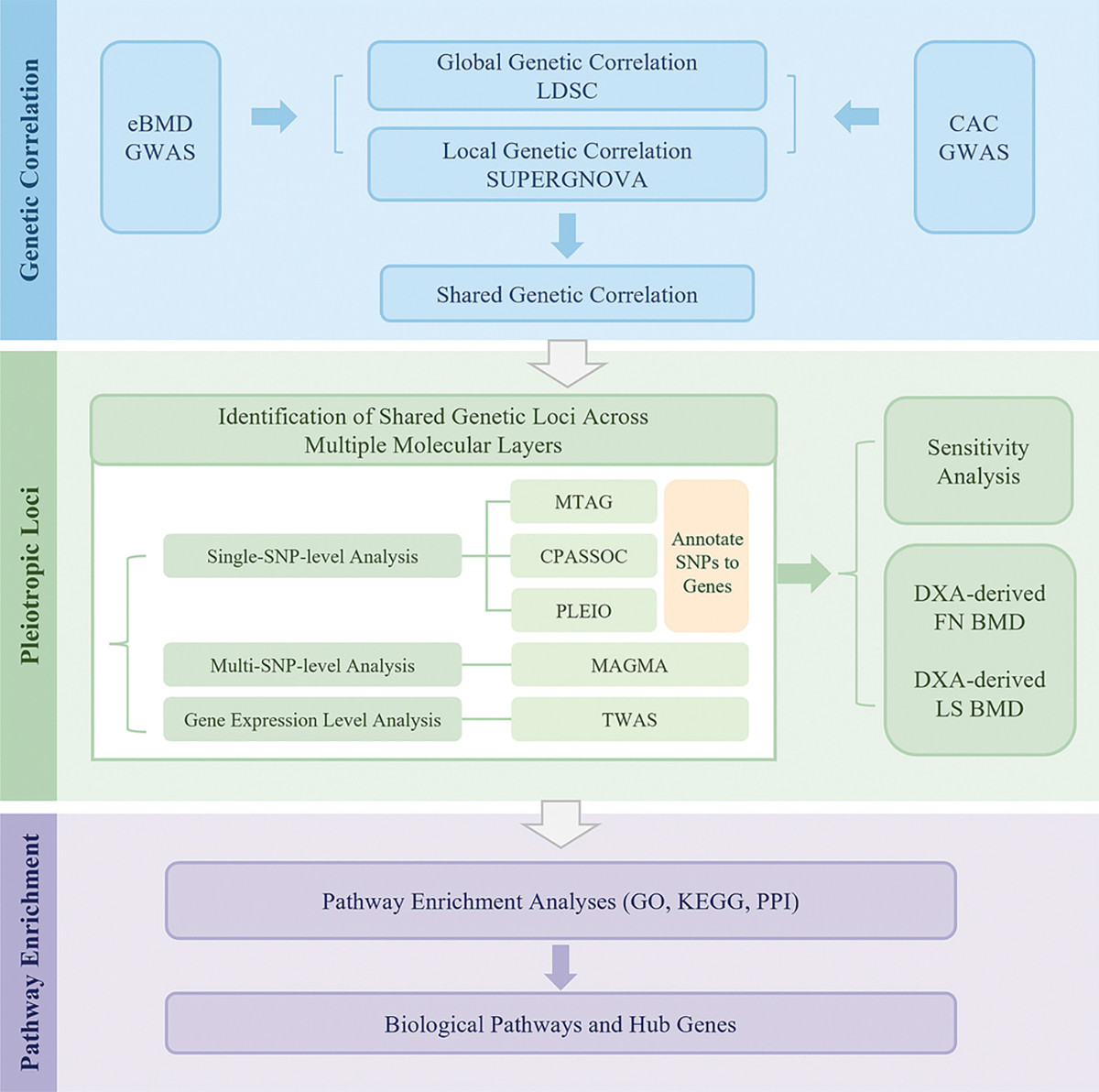
Flow-chart of the overall study design. GWAS: genome-wide association study; CAC: coronary artery calcification; eBMD: estimated calcaneal bone mineral density; LDSC: linkage disequilibrium score regression; MTAG: multi-trait analysis of GWAS; CPASSOC: Cross-Phenotype Association; PLEIO: Pleiotropic Locus Exploration and Interpretation using Optimal test; MAGMA: Multi-marker Analysis of GenoMic Annotation; TWAS: transcriptome-wide association study; DXA: dual-energy X-ray absorptiometry; BMD: bone mineral density; FN: femoral neck; LS: lumbar spine; PPI: protein-protein interaction; GO: Gene Ontology; KEGG: Kyoto Encyclopedia of Genes and Genomes.

Therefore, we aim to provide novel insights into the biological mechanisms underlying the calcification paradox through exploring the pleiotropic associations and shared genetic signals linking CAC and estimated calcaneal bone mineral density (eBMD). Identifying shared genetic architecture may not only offer clues to common etiological mechanisms but also facilitate the development of biomarkers and therapeutic strategies. To this end, we adopted a multi-layered analytical framework (Fig. 1). First, genome-wide and local genetic correlations were evaluated to identify the shared genetic landscape. Second, multiple methods were employed to identify genetic variants and genes driving this association across different molecular levels. This included a single-nucleotide polymorphism (SNP) level analysis, a multi-SNP level analysis, and a gene expression level analysis. To enable a comprehensive functional interpretation, SNPs identified from the cross-trait meta-analyses were annotated to their putative effector genes. Sensitivity analyses using DXA-derived femoral neck (FN) and lumbar spine (LS) BMD (instead of eBMD) validated the robustness of key loci. Finally, all candidate genes identified across different molecular layers were integrated for pathway enrichment analyses to explore the underlying biological mechanisms.

## 2. Materials and methods

### 2.1. GWAS data availability

The hitherto largest genome-wide association study (GWAS) meta-analysis of CAC was conducted by the Cohorts for Heart and Aging Research in Genomic Epidemiology (CHARGE) Consortium and other collaborating cohorts, involving 26,909 individuals of European ancestry [9]. CAC was evaluated by calculating the Agatston score based on the amount of plaque observed in noninvasive cardiac computed tomography scans. This GWAS identified 16 significant lead SNPs in 11 independent genomic risk loci (*P* < 5.0 × 10^−8^).

The hitherto largest GWAS of eBMD was conducted by Morris et al., comprising 426,824 individuals of European ancestry from the UK Biobank [10]. Estimated calcaneal bone mineral density was measured using heel quantitative ultrasound. eBMD benefits from a significantly larger GWAS sample size and high heritability, enhancing the statistical power and precision of predicting osteoporotic fractures, which allows for more robust and reliable results in population-based studies. This GWAS identified 1103 conditionally independent genetic variants mapping to 515 loci (*P* < 6.6 × 10^−9^).

### 2.2. Genetic correlation analysis

To estimate the global genetic correlation between CAC and eBMD, cross-trait linkage disequilibrium score regression (LDSC) was employed [11]. Genetic correlation (*r*_*g*_) represents the average genetic association between two traits, with values ranging from −1 to 1.

We evaluated the local genetic correlation using SUPERGNOVA, an algorithm that quantifies the genetic similarity of two traits across 2353 approximately independent genomic regions [12]. Genetic covariance (*ρ*) represents the local correlation coefficient between two traits, quantifying the extent and direction of the shared genetic effects within a given genomic region. A Bonferroni-corrected P-threshold of 0.05/2353 was used to define statistical significance. We drew a rectangular-Manhattan plot using the R language CMplot package [13].

### 2.3. Cross-trait meta-analysis and colocalization analysis (single-SNP-level analysis)

To investigate whether the genetic variants had independent effects on both traits, we performed a cross-trait meta-analysis using multi-trait analysis of GWAS (MTAG) [14], Cross-Phenotype Association (CPASSOC) [15], and Pleiotropic Locus Exploration and Interpretation using Optimal test (PLEIO) [16]. MTAG employs inverse-variance-weighted meta-analysis to combine single-trait GWAS data, generating trait-specific effect estimates for each SNP. CPASSOC, on the other hand, improves statistical power by accounting for heterogeneity effects across traits, refining the SNP effect estimates. PLEIO further optimizes statistical power by additionally considering the environmental correlations from the GWAS data. Collectively, these phenotype association tests leverage distinct algorithms to identify SNP effect estimates based on GWAS summary statistics, with shared genetic variants being identified and selected based on predefined significance thresholds.

For MTAG, significant lead SNPs for each trait were tagged by PLINK based on the following criteria: –clump-p1 5e-8, –clump-p2 1e-5, –clump-r2 0.1, –clump-kb 500 [17]. Index SNPs were selected (*P* < 5 × 10^−8^), and SNPs with *P* < 1 × 10^−5^, in linkage disequilibrium (LD) (*r*^2^ ≥ 0.1), and within 500 kb of each index SNP were clumped [18]. Subsequently, an index SNP for each LD block was selected as a lead SNP, ensuring that all lead SNPs were independent and significant. We then identified lead SNP pairs (one SNP from each trait) that were located within 500 kb of each other. For these paired SNPs, we calculated their LD. If *r*^2^ ≥ 0.8, they were classified as shared loci [19].

For CPASSOC and PLEIO, we used the PLINK clumping function to target independent pleiotropic SNPs: a significance threshold for index SNPs of *P* < 5 × 10^−8^ (–clump-p1 5e-8), a secondary significance threshold for clumped SNPs of *P* < 1 × 10^−5^ (–clump-p2 1e-5), a minimum *r*^2^ threshold of 0.2 (–clump-r2 0.2) and a maximum distance of 500 kb (–clump-kb 500) [17]. Significant index SNPs were recognized with *P*CPASSOC/PLEIO <5 × 10^−8^ and *P*single-trait <1 × 10^−3^. Four groups of these significant index SNPs were further categorized based on their single-trait and cross-trait characteristics, including “known” shared SNPs, “single-trait-driven” shared SNPs, “LD-tagged” shared SNPs and “novel” shared SNPs [20]. Among them, the “novel” shared SNPs were of most interest, defined as those that were neither significant in both traits nor in LD (*r*^2^ < 0.2 or kb < 500) with reported significant SNPs.

To determine whether the shared variants are causally responsible for two GWAS signals, we performed a colocalization analysis using COLOC; if the posterior probability for H4 (PPH4) of the shared locus exceeded 0.70, this locus was colocalized [21].

We used Ensembl Variant Effect Predictor (VEP) [22] and 3DSNP [23] for gene annotation of the index SNPs.

### 2.4. MAGMA analysis (multi-SNP-level analysis)

We applied Multi-marker Analysis of GenoMic Annotation (MAGMA) to perform multi-SNP-level analysis, annotating SNPs at the gene-wide level and quantifying the association of all markers within a gene and a phenotype [24]. In this analysis, we applied the SNP-wise mean model. Significant genes (*P* < 1 × 10^−3^) from both traits were merged to identify shared genes. These significant shared genes were further classified according to whether they had been previously reported to be associated with traits related to either CAC or BMD.

### 2.5. Transcriptome-wide association study (gene expression level)

To identify associations between transcriptome gene expression in specific tissues and traits, we conducted a transcriptome-wide association study (TWAS) using FUSION based on expression weights from 49 GTEx (Genotype-Tissue Expression, version 8) tissues [25]. A Bonferroni correction was applied within each tissue to account for multiple comparisons. Significantly associated genes (*P*_Bonferroni_ < 0.05) from both traits were merged to identify shared gene expressions.

### 2.6. Sensitivity analysis

We noticed a pronounced sample size imbalance between the CAC GWAS (~27,000 individuals) and the eBMD GWAS (~427,000 individuals), which may introduce statistical bias, potentially inflating type I error rates and leading to an enrichment of eBMD-driven loci in cross-trait meta-analyses. Moreover, eBMD was measured by heel ultrasound rather than by DXA, limiting its clinical interpretability and generalizability of findings. To address these limitations, we further conducted a sensitivity analysis using DXA-derived GWAS summary statistics of FN BMD (*N* = 49,988) and LS BMD (*N* = 44,731) [26], which also showed comparable sample sizes to the CAC GWAS. Significant loci or genes derived from the main analysis were replicated by going through the same set of multi-layered pleiotropy analytical strategy, but using GWAS summary statistics of FN and LS BMD. Significance thresholds were set as nominal *P* < 0.05.

### 2.7. Pathway enrichment analyses

To further analyze the functions of significant shared genes identified through cross-trait analysis, MAGMA, and TWAS, we performed pathway enrichment analyses, including protein-protein interaction (PPI) network, Gene Ontology (GO) [27,28] and Kyoto Encyclopedia of Genes and Genomes (KEGG) [29] analysis. To construct a PPI network, all significant shared genes were entered into the STRING database (version 12.0, https://string-db.org/) [30]. Cytoscape software (version 3.7.1) was used to determine hub genes and visualize the network [31]. GO annotates genes in three biological aspects: biological process, cellular component, and molecular function. KEGG reveals the molecular functions of biological systems. We conducted GO and KEGG analyses using the R language clusterProfiler package [32], with *P* < 0.05 as statistical significance.

Detailed information is available in the Supplementary Methods.

## 3. Results

### 3.1. Genetic correlation analysis

**Fig. 2.**
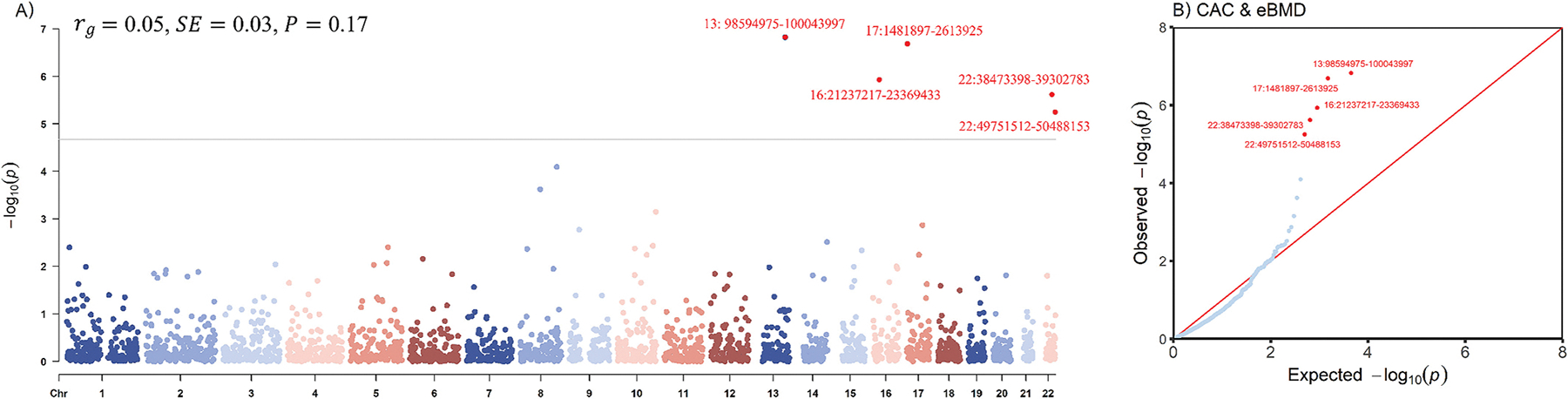
Global and local genetic correlations between CAC and eBMD. A) Manhattan-style plot shows the local genetic correlation between CAC and eBMD using SUPERGNOVA, with the global genetic correlation from LDSC displayed in the upper left. Red dots represent significant loci after multiple testing adjustment (*P* < 0.05/2353). B) QQ plot presents region-specific *P*-values from the local genetic correlation between CAC and eBMD. *r_g_*: genetic correlation; *SE*: standard error; CAC: coronary artery calcification; eBMD: estimated calcaneal bone mineral density. (For interpretation of the references to colour in this figure legend, the reader is referred to the web version of this article.)

We found no evidence of a significant global genetic correlation between CAC and eBMD (*r*_*g*_ = 0.05, *P* = 0.17). When the whole genome was partitioned, five significant regions (13q32.2, 16p12.2, 17p13.3, 22q13.1, 22q13.3) were observed (Fig. 2). The most significant signal was at 13q32.2 (chr13:98594975–100,043,997, *ρ* = −0.0014, *P* = 1.50 × 10^−7^), a region that was not previously reported for CAC or eBMD. Notably, this locus harbors *FARP1*, a gene linked to coronary artery disease (CAD), and *STK24*, which has been implicated in vascular development of endothelial cells [33,34]. The second most significant signal was at 17p13.3 (chr17:1481897–2,613,925, *ρ* = 0.0023, *P* = 2.06 × 10^−7^), which harbors *SMG6*, a gene previously reported to be associated with both CAD [35] and eBMD [10]. In addition, regions 16p12.2 (chr16:21237217–23,369,433, *ρ* = 0.0007, *P* = 1.17 × 10^−6^), 22q13.1 (chr22:38473398–39,302,783, *ρ* = 0.0006, *P* = 2.41 × 10^−6^), and 22q13.3 (chr22:49751512–50,488,153, *ρ* = 0.0006, *P* = 5.64 × 10^−6^) were only reported to be associated with eBMD according to previous literature [10].

### 3.2. Cross-trait meta-analysis and colocalization analysis

MTAG identified 9 significant lead SNPs for CAC and 2036 for eBMD (Table S1). We identified two CAC lead SNPs mapped within 500kb of the eBMD lead SNPs, forming several lead SNP pairs (Table S2). Among these, only one pair, located at 17p13.3/*SMG6*, showed not only physical proximity but also a significant LD (*r*^2^ ≥ 0.8) and therefore represented a shared signal.

**Fig. 3.**
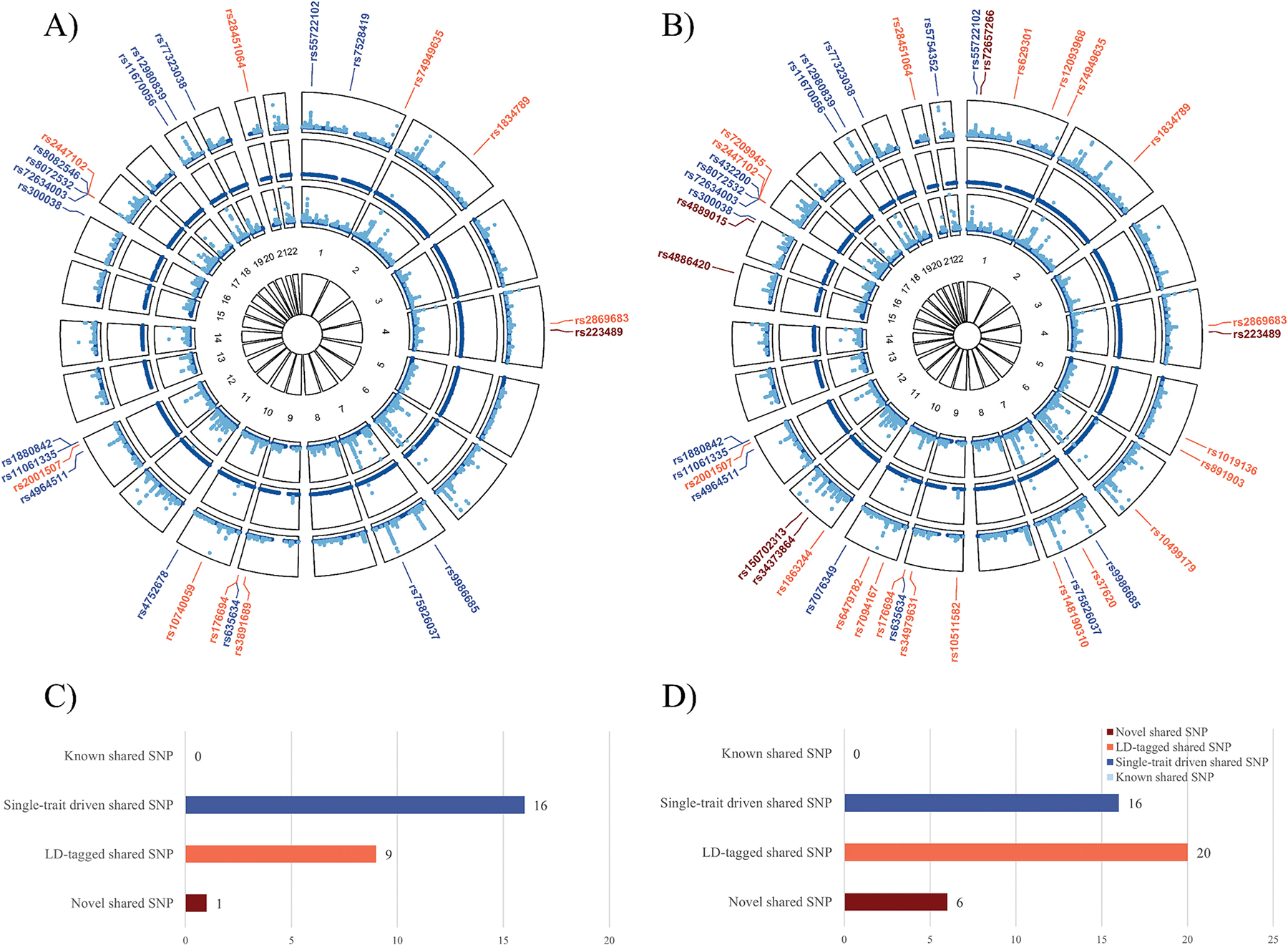
Cross-phenotype associations between CAC and eBMD. In each circular Manhattan plot, the outermost circle shows the cross-trait meta-analysis results between CAC and eBMD estimated by A) CPASSOC method, B) PLEIO method. From periphery to center after the outermost cross-trait meta-analysis, each circle shows the GWAS results for CAC and then for eBMD. The light blue indicates variants with genome-wide significance (*P*_CAC_ < 5 × 10^−8^ or *P*_eBMD_ < 6.6 × 10^−9^), while the dark blue indicates variants not reaching genome-wide significance. SNPs are classified into four categories based on their single-trait and cross-trait characteristics: (1) “known” shared SNPs, which are significant in both traits (*P*_*CAC*_ < 5 × 10^−8^ and *P*_*eBMD*_ < 6.6 × 10^−9^) with *P*_CPASSOC/PLEIO_ < 5 × 10^−8^; (2) “single-trait-driven” shared SNPs, which are significant in only one trait (either *P*_*CAC*_ < 5 × 10^−8^ or *P*_*eBMD*_ < 6.6 × 10^−9^) with *P*_CPASSOC/PLEIO_ < 5 × 10^−8^; (3) “LD-tagged” shared SNPs, which are not significant in either trait (*P*_*CAC*_ ≥ 5 × 10^−8^ and *P*_*eBMD*_ ≥ 6.6 × 10^−9^) with *P*_CPASSOC/PLEIO_ < 5 × 10^−8^, but are in LD (*r*^2^ ≥ 0.2) or in the vicinity (±500kb) of significant SNPs reported by single-trait GWAS; (4) “novel” shared SNPs, which are not significant in both traits but have *P*_CPASSOC/PLEIO_ < 5 × 10^−8^, and are not in LD with index SNPs identified by single-trait GWAS. These four categories of SNPs are represented in light blue, dark blue, orange, and brown, respectively, with their RS IDs listed around the circles. C) Bar plot of significant pleiotropic loci between CAC and eBMD estimated by CPASSOC. D) Bar plot of significant pleiotropic loci between CAC and eBMD estimated by PLEIO. CAC: coronary artery calcification; eBMD: estimated calcaneal bone mineral density; GWAS: genome-wide association study; LD: linkage disequilibrium; SNP: single-nucleotide polymorphism; CPASSOC: Cross-phenotype association; PLEIO: Pleiotropic Locus Exploration and Interpretation using Optimal test. (For interpretation of the references to colour in this figure legend, the reader is referred to the web version of this article.)

CPASSOC identified 26 significant pleiotropic SNPs, while PLEIO identified 42 significant SNPs (Fig. 3 and Table S3). These significant SNPs collectively mapped to 190 non-duplicated genes using PLINK, VEP, and 3DSNP, which were included in the subsequent pathway enrichment analyses. Among all significant SNPs, 21 were identified by both methods, and 4 additional SNPs identified by CPASSOC were in high LD with SNPs identified by PLEIO. Of note, the top three significant loci (rs8072532, rs8082546/rs432200, rs72634003) were all located at 17p13.3, mapping to their linear closest gene *SMG6*. Specifically, the most significant locus identified by both methods was rs8072532 (*P*CPASSOC = 1.71 × 10^−159^, *P*PLEIO = 1.43 × 10^−229^). The second most significant locus identified by CPASSOC (rs8082546, *P* = 1.19 × 10^−44^) or PLEIO (rs432200, *P* = 3.04 × 10^−63^) were in high LD (*r*^2^ = 0.98). The third most significant locus identified by both methods was rs72634003 (*P*CPASSOC = 4.39 × 10^−44^, *P*PLEIO = 3.83 × 10^−61^).

As for novel shared SNPs, CPASSOC identified one (rs223489) while PLEIO identified six (rs72657266, rs223489, rs150702313, rs34373864, rs4886420, rs4889015). SNP rs223489 was mapped to *MANBA* and *UBE2D3*. *MANBA* is associated with coronary heart disease (CHD) [36], while *UBE2D3* has been predicted as a potential therapeutic target in CAD [37].

We then determined whether significant shared SNPs were colocalized. The significant lead SNP pair identified by MTAG was colocalized. Of the 26 significant shared SNPs identified by CPASSOC, 11 (42.3 %) showed a PPH4 > 0.7. Among the 42 significant SNPs identified by PLEIO, 12 (28.6 %) showed a PPH4 > 0.7. The top three most significant SNPs determined by both methods (rs8072532, rs8082546/rs432200, and rs72634003), as well as the novel co-discovered SNP (rs223489), were all colocalized.

### 3.3. MAGMA analysis

As an effective complement to individual variant analysis, we used MAGMA for gene-based analysis, identifying 89 significant genes for CAC and 3573 for eBMD (Table S4-S5). After merging these significant genes, we identified 27 shared genes, which were included in the subsequent pathway enrichment analyses. These 27 genes were further divided into three categories according to whether they had been reported: 4 were reported by existing literature as associated with both traits *(PRRX1*, *MRAS*, *ARID5B*, *SMG6*), 17 were reported to be linked to only one trait (*ANXA9*, *VAMP5*, *VAMP8*, *EBF1*, *EPDR1*, *ZFPM2*, *BTBD16*, *ODAD2*, *MPP7*, *FOSL1*, *ADGRD1*, *MMP14*, *CCDC97*, *FBXO46*, *RSPH6A*, *ZBED4*, *ALG12*), and 6 were novel (*MINDY1*, *FIP1L1*, *RCL1*, *MRPL52*, *PAFAH1B1*, *CRELD2*) (Table S6). The four genes associated with both traits have been identified as significant loci in previous GWAS. Specifically, these genes were reported in eBMD GWAS, with *PRRX1* linked to aortic valve calcification, *MRAS* and *SMG6* to coronary atherosclerosis, and *ARID5B* to CAC [9,10,38–40]. Functional studies have validated the roles of *PRRX1* in inhibiting osteoblast differentiation and *ARID5B* in promoting chondrogenesis and vascular smooth muscle calcification [9,41,42].

Notably, *SMG6* was also detected by MTAG, CPASSOC, and PLEIO. Additionally, *PAFAH1B1*, located also at 17p13.3, was also identified by PLEIO.

### 3.4. Transcriptome-wide association study

To further investigate the relationship between transcriptome-wide gene expression and traits, we performed TWAS and identified three independent gene-tissue pairs, namely *PAFAH1B1*, *SMG6*, and *IGFBP3*, enriched in the tibial nerve, fibroblasts, and heart, respectively (Table S7). *SMG6* and *PAFAH1B1* were also identified as shared genes in our cross-trait analysis and by MAGMA. *IGFBP3* was reported to be associated with CAC, suggesting a causal role in both early and advanced atherosclerosis through its direct impact on smooth muscle cell calcification, proliferation, and migration [9].

### 3.5. Sensitivity analysis

The sensitivity analysis using FN and LS BMD GWAS confirmed the robustness of key findings. In CPASSOC and PLEIO analyses, significant pleiotropic SNPs mapped to *SMG6* were consistently identified by using data of FN BMD (Table S8). Similarly, MAGMA analysis confirmed significant associations of both *SMG6* and *PAFAH1B1* by using data of FN BMD (Table S9). Furthermore, TWAS analysis consistently identified *SMG6* and *PAFAH1B1* as significant pleiotropic genes (Table S10). Collectively, these results supported the pleiotropic roles of key genes *SMG6* and *PAFAH1B1* across different skeletal sites.

### 3.6. Pathway enrichment analyses

**Fig. 4.**
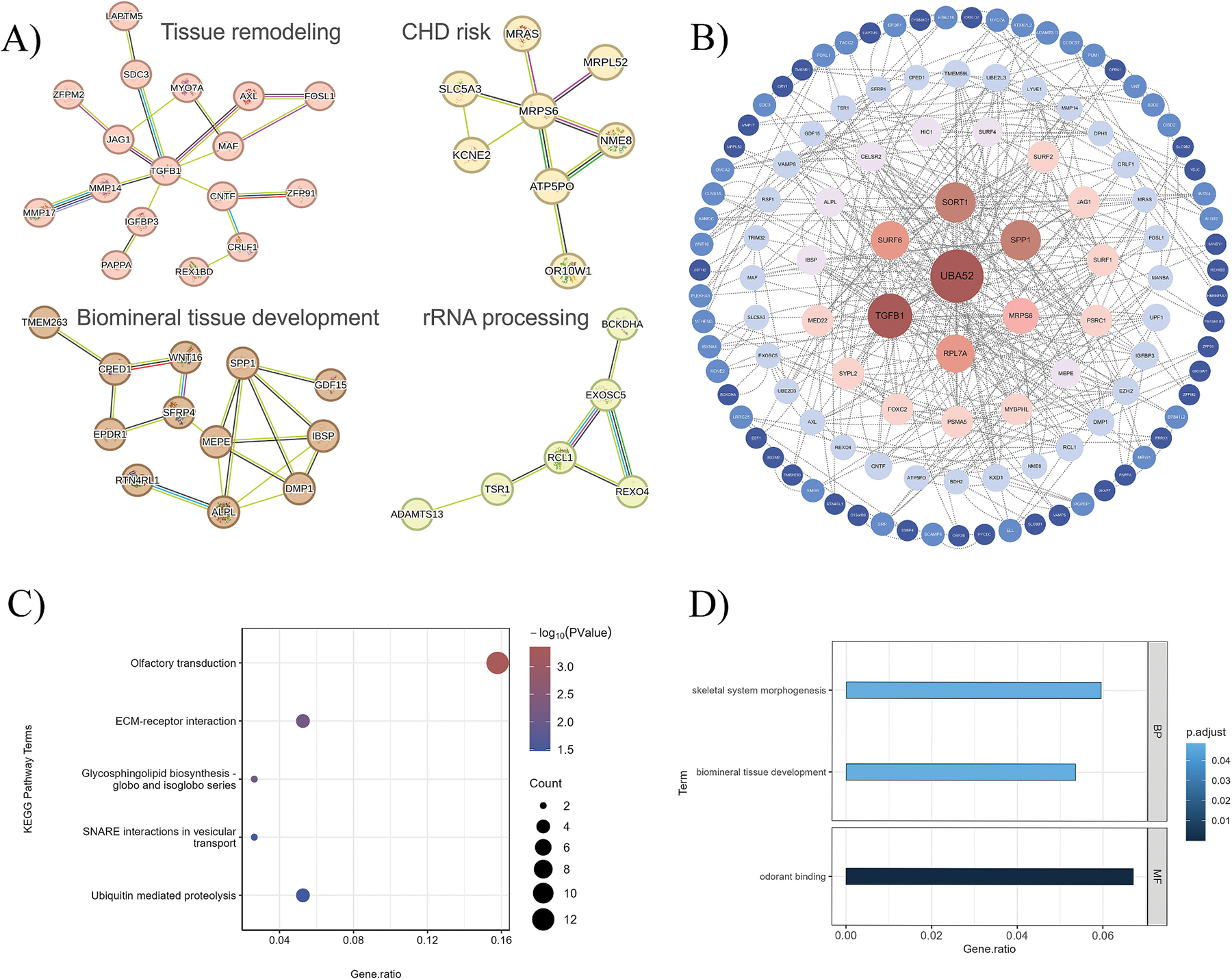
GO, KEGG, and PPI network analyses. A) Significant clusters in the PPI network analysis using the STRING database. B) Schematic representation of hub genes screened by the degree in the PPI network. The node size and colour are positively correlated with degree: brown indicates a larger degree, and blue indicates the minimum degree. C) KEGG pathway enrichment analysis without multiple testing adjustment. D) GO biological function enrichment analysis after FDR correction. PPI, protein-protein interaction; GO, Gene Ontology; KEGG, Kyoto Encyclopedia of Genes and Genomes; BP, biological process; MF, molecular function. (For interpretation of the references to colour in this figure legend, the reader is referred to the web version of this article.)

To provide a comprehensive view of potential biological mechanisms, we included all significant genes identified from the CAC-eBMD-associated analyses for pathway exploration. Pathway enrichment analyses, including PPI network, GO, and KEGG analyses, were performed on 211 non-duplicated significant shared genes identified by the cross-trait analysis (*N* = 190), MAGMA (*N* = 27), or TWAS (*N* = 3). The PPI network comprised 173 nodes and 189 edges (Table S11). Significantly enriched clusters included biomineral tissue development, tissue remodeling, coronary heart disease (CHD) risk loci, and rRNA processing (Fig. 4A). We further screened the hub genes by their degrees in the PPI network (Fig. 4B) and identified four top genes, namely, *UBA52* (degree = 32), *TGFB1* (degree = 24), *SORT1* (degree = 20), and *SPP1* (degree = 20). KEGG pathway analysis revealed enrichment primarily in olfactory transduction, ECM-receptor interaction, glycosphingolipid biosynthesis, SNARE interactions in vesicular transport, and ubiquitin-mediated proteolysis (Fig. 4C). The significant biological processes for GO analysis were biomineral tissue development and skeletal system morphogenesis, while the significant molecular function for GO analysis was odorant binding (Fig. 4D). These results suggested that the identified genes may simultaneously regulate both skeletal and vascular calcification through shared pathways, providing insights into the common mechanisms underlying the calcification paradox. Full results of KEGG and GO enrichment analyses are provided in Tables S12-S13.

## 4. Discussion

To our knowledge, this is the first study that extensively investigates the genetic pleiotropy underlying CAC and eBMD. Using the largest available genome-wide summary statistics, we identified shared genetic architectures at single-SNP, multi-SNP, and gene expression levels. The co-regulated genes and their enriched biological pathways provide critical insights into the CAC-BMD phenotypic association, also known as the calcification paradox. Notably, our findings highlight shared genetic signals on chromosome 17, particularly at the loci *SMG6* and *PAFAH1B1*, both of which were consistently captured and validated by multiple methods.

Although no significant global genetic correlation was observed between CAC and eBMD, regional analysis revealed several loci with significant positive or negative genetic covariance. This pattern suggested a complex genetic architecture where the non-significant global correlation reflected a balance of opposing local effects rather than a lack of shared genetics. Therefore, integrating global and local approaches was essential for uncovering biologically meaningful shared loci. Our analysis identified 17p13.3 as a key pleiotropic locus, which is consistent with the role of chromosome 17 in both cardiovascular disease and bone health. The 17p11 and 17q21 regions are linked to myocardial infarction [43]. The *SOST* gene on 17q21 encodes sclerostin, a protein that regulates bone formation [44]. The 17p13 locus has been identified as significant in previous GWAS of CAD or BMD [35,45]. While previous studies primarily focused on each phenotype individually, our findings provide evidence that 17p13 functions as a shared locus, potentially linking the mechanisms of vascular and skeletal calcification.

To elucidate the calcification paradox at the level of molecular mechanisms, we employed various cutting-edge statistical genomics approaches to identify shared pleiotropic loci. The primary analysis, leveraging the large-scale eBMD GWAS, identified a comprehensive set of 211 candidate genes. Specifically, cross-trait analysis revealed 190 shared genes, MAGMA identified 27 shared genes, and TWAS determined 3 independent gene-tissue pairs. Collectively, these common genetic architectures underscore the widespread pleiotropy across CAC and eBMD. Among them, *SMG6* and *PAFAH1B1* showed consistent significance across multi-layered analysis. Recognizing the potential bias of eBMD data, our subsequent sensitivity analysis used the clinical gold-standard DXA measurement to test the robustness of highlighted candidate genes. The consistent validation of both *SMG6* and *PAFAH1B1* loci confirmed the robustness and clinical relevance of results. *SMG6* has been confirmed as a susceptible locus for CAD [35] and BMD [45], with both associations independently replicated in previous GWASs. The key contribution of our study was the co-detection of this locus within a unified pleiotropic framework. Furthermore, while *SMG6* was not genome-wide significant in CAC GWAS, our detection suggested that its pathogenic effect may emerge during subclinical atherosclerosis, prior to clinically evident CAD. These findings implied *SMG6* as a potential upstream regulator in the calcification paradox and a candidate therapeutic target for early intervention. Functionally, *SMG6* encodes a ribonuclease essential for nonsense-mediated mRNA decay (NMD), a crucial mRNA quality control system that maintains cellular homeostasis [46,47]. Although direct evidence between NMD and skeletal or vascular mineralization is currently limited, the fundamental role of *SMG6* in cellular homeostasis suggests a plausible pleiotropic mechanism. *PAFAH1B1*, located in the same region, regulates cell survival and differentiation of osteoclast precursors during osteoclastogenesis [48], implying a possible involvement in bone remodeling.

Given the substantially larger sample size of eBMD GWAS and the successful replication of key loci using the DXA-based datasets, we integrated all significant CAC-eBMD genes for downstream pathway enrichment. This strategy allowed us to generate a broad overview of potential biological pathways connecting vascular calcification and bone density. Our results revealed oxidative stress and the ubiquitin-proteasome system (UPS) as critical biological components connecting the two degenerative processes. Reactive oxygen species (ROS) are important for osteochondrogenic trans-differentiation of vascular smooth muscle cells [5], while ROS-induced oxidative stress increases bone resorption [49]. Crucially, both ROS and UPS may converge on *RUNX2*, a crucial transcription factor essential for osteoblast differentiation and vascular calcification [50,51]. Elevated ROS induces degradation of *RUNX2*, while UPS plays a role in its posttranslational regulation [52,53]. Therefore, the dysregulation of these mechanisms may disrupt *RUNX2* stability, leading to impaired bone formation and pathological vascular calcification. In addition, the four PPI hub genes demonstrate their pivotal roles in both vascular and skeletal health. *UBA52* encodes ubiquitin, reinforcing the importance of the UPS pathway revealed in our enrichment analyses [54]. *TGFB1* encodes TGF-β1, which controls osteoblast and osteoclast differentiation, thereby balancing bone formation and resorption [55]. TGF-β1 also associates with collagen secretion and activation in myocardial fibroblasts involved in cardiac fibrosis [56]. *SPP1* encodes osteopontin, a key regulator in bone remodeling that promotes joint and cartilage destruction [57]. *SPP1* also calcifies atherosclerotic plaques by promoting fatty streak formation and plaque development [58]. *SORT1* encodes sortilin, involved in the formation of microcalcifications in smooth muscle cell culture by facilitating its recruitment to extracellular vesicles [59].

Our study presents a comprehensive investigation into the pleiotropic associations between CAC and BMD, offering several public health and clinical implications, particularly within the context of European populations. First, our findings offer a genetic explanation for the calcification paradox. Since previous studies demonstrated an absence of causal association, we suggested that the observed phenotypic association might result from horizontal pleiotropy. This insight reframes the biological mechanism from a simple causal effect model (e.g., bone calcium loss leading to vascular deposition) to a complex network of shared molecular pathways. Second, the identified pleiotropic loci may help prioritize genes for further experimental validation and therapeutic exploration, especially those consistently identified across different skeletal sites (e.g., *SMG6*, *PAFAH1B1*), although their relevance must be confirmed across diverse ancestries and between sexes. Third, while requiring substantial further validation, the shared genetic basis supports the potential for simultaneous prevention or co-treatment of osteoporosis and vascular calcification. For instance, measuring thoracic BMD through CAC scans [60] or measuring spine BMD using DXA, accompanied by a simple lateral vertebral assessment with vascular calcification scoring [61], may allow early detection of osteopenia and osteoporosis. Such approaches may improve clinical efficiency, reduce unnecessary radiation exposure, and guide timely interventions. However, these translational implications remain preliminary. Further experimental and clinical studies incorporating diverse ancestral backgrounds and sex-stratified analyses are needed to determine the interpretability of clinical protocols and therapeutic strategies.

Several limitations also need to be acknowledged. First, due to the limitations of available GWAS data, our analyses were restricted to a European ancestry population and lacked sex-specific assessments, thereby limiting the generalizability of our findings. Second, although additional sensitivity analyses using DXA-derived FN and LS BMD partially addressed site-specific concerns, further studies are needed to improve the clinical interpretability and generalizability of findings. Third, the co-regulated genes and associated biological pathways identified in our study required further validation through in vitro or in vivo experiments to elucidate their precise molecular functions. Finally, while the original CAC GWAS implemented standardized measurement protocols and statistical quality control to minimize inter-cohort heterogeneity, unavoidable differences in scanner models and parameters across cohorts may still introduce residual heterogeneity in CAC measurements.

## 5. Conclusion

In conclusion, this study provides valuable insights into the horizontal genetic pleiotropy underlying CAC and eBMD, highlighting shared genetic and biological mechanisms that influence both cardiovascular and bone health. By examining shared loci across multi-layers, our results indicate a comprehensive shared genetic architecture with a special focus on chromosome 17, particularly on loci *SMG6* and *PAFAH1B1*. Our work advances understanding of the molecular basis underpinning calcification paradox and opens new avenues for the simultaneous monitoring and management of osteoporosis and cardiovascular disease.

## Supplementary Material

Supplementary materials

## Data Availability

CAC GWAS summary statistics are publicly available from https://cvd.hugeamp.org/. eBMD GWAS summary statistics are publicly available from http://www.gefos.org/.

## Appendix A. Supplementary data

Supplementary data to this article can be found online at https://doi.org/10.1016/j.bone.2025.117719.

## Abbreviations

BMD: bone mineral density
MR: Mendelian randomization
CAC: coronary artery calcification
DXA: dual-energy X-ray absorptiometry
eBMD: estimated calcaneal bone mineral density
SNP: single-nucleotide polymorphism
FN: femoral neck
LS: lumbar spine
GWAS: genome-wide association study
CHARGE: Cohorts for Heart and Aging Research in Genomic Epidemiology
LDSC: linkage disequilibrium score regression
MTAG: multi-trait analysis of GWAS
CPASSOC: Cross-Phenotype Association
PLEIO: Pleiotropic Locus Exploration and Interpretation using Optimal test
LD: linkage disequilibrium
VEP: Ensembl Variant Effect Predictor
MAGMA: Multi-marker Analysis of GenoMic Annotation
TWAS: transcriptome-wide association study
PPI: protein-protein interaction
GO: Gene Ontology
KEGG: Kyoto Encyclopedia of Genes and Genomes
CAD: coronary artery disease
CHD: coronary heart disease
NMD: nonsense-mediated mRNA decay
UPS: ubiquitin-proteasome system
ROS: Reactive oxygen species
