# Supplementary material for: A comprehensive multi-layered analysis reveals genetic pleiotropy underlying coronary artery calcification and bone mineral density
Source: Bone. Author manuscript; Available in PMC 2026 Feb 23. (PMC12928696; doi:10.1016/j.bone.2025.117719)
Supplement: Supplementary materials [file NIHMS2139860-supplement-Supplementary_materials.zip › 1-s2.0-S875632822500331X-mmc1.docx]

**Supplementary Methods**

**GWAS data availability**

The hitherto largest genome-wide association study (GWAS) meta-analysis of coronary artery calcification **(CAC)** was conducted by the Cohorts for Heart and Aging Research in Genomic Epidemiology (CHARGE) Consortium and other collaborating cohorts^(1)^. This study utilized an inverse variance weighted meta-analysis to combine summary statistics from 16 cohorts, encompassing a total of 26,909 individuals of European ancestry and 8,867 individuals of African ancestry. Only the GWAS summary statistic from the European ancestry was included in this study to avoid the potential bias resulting from population structure. To ensure comparability, a standardized approach was employed, wherein all cohorts quantified CAC using computed tomography based on the standardized Agatston scoring method^(2)^. To minimize potential heterogeneity arising from different scanners and acquisition settings, the original GWAS applied a quality control framework, including regression analyses with adjustment for age, sex, and principal components, followed by an inverse variance weighted meta-analysis to combine the results. This GWAS identified 16 significant lead SNPs in 11 independent genomic risk loci, eight of which were novel for CAC. The genome-wide significance threshold was *P* < 5.0×10^−8^.

The hitherto largest GWAS of estimated calcaneal bone mineral density **(eBMD)** was conducted by Morris et al., comprising 426,824 individuals of European ancestry from the UK Biobank^(3)^. Heel bone quality was assessed using quantitative ultrasound, based on the speed of sound and broadband ultrasound attenuation, to provide a robust assessment of bone density. eBMD benefits from a significantly larger GWAS sample size and high heritability, enhancing the statistical power and precision of predicting osteoporotic fractures, which allows for more robust and reliable results in population-based studies. This GWAS applied approximate conditional and joint genome-wide association analysis to identify 1,103 conditionally independent genetic variants reaching genome-wide significance (*P* < 6.6×10^−9^), of which 423 were novel. These variants mapped to 515 loci, including 301 novel loci. The complete set of summary statistics for eBMD was used.

**Statistical analysis**

**Genetic correlation analysis**

To estimate the global genetic correlation between CAC and eBMD, cross-trait linkage disequilibrium score regression (**LDSC**) was employed^(4)^. This method is based on the principle that SNPs with high linkage disequilibrium (LD) exhibit higher statistical values on average than those with low LD. The resulting genetic correlation ($r_{g}$) represents the average association between two traits, with values ranging from -1 to 1. Positive or negative values indicate the direction of the association, and values closer to 1 indicate a pronounced correlation.

To quantify the genetic similarity of two traits in specific genomic regions, we further evaluated the local genetic correlation between CAC and eBMD using **SUPERGNOVA**, an algorithm that quantifies the genetic similarity across 2,353 approximately independent genomic regions^(5)^. Genetic covariance ($\rho$) represents the local correlation coefficient between two traits, reflecting the magnitude and direction of their shared genetic effects within each genomic region. Positive $\rho$ indicates concordant genetic effects, whereas negative $\rho$ reflects opposing genetic influences. A Bonferroni-corrected P-threshold of 0.05/2353 was used to define statistical significance. We drew a rectangular-Manhattan plot using the R language CMplot package^(6)^.

**Cross-trait meta-analysis and colocalization analysis (single-SNP-level analysis)**

To investigate whether the genetic variants had independent effects on both CAC and eBMD, we performed a cross-trait meta-analysis using three methods: multi-trait analysis of GWAS (**MTAG**)^(7)^, Cross-Phenotype Association (**CPASSOC**)^(8)^ and Pleiotropic Locus Exploration and Interpretation using Optimal test (**PLEIO**)^(9)^. Collectively, these phenotype association tests leverage distinct algorithms to identify SNP effect estimates based on GWAS summary statistics, with shared genetic variants being selected based on predefined significance thresholds.

The MTAG analysis utilizes genetic correlation between traits to enhance the power to detect the association in genome-wide analyses, generalizing inverse-variance-weighted meta-analysis of GWAS summary statistics across different traits to generate trait-specific effect estimates for each SNP^(7)^. For each trait, significant lead SNPs were tagged using PLINK based on the following criteria: --clump-p1 5e-8, --clump-p2 1e-5, --clump-r2 0.1, --clump-kb 500^(10)^. Index SNPs were selected by ranking smallest to largest *P* value (still with a cut-off value of *P* < 5×10⁻⁸). First, the most significant index SNP was selected as the lead SNP, and all SNPs with *P* < 1×10⁻⁵, in LD ($r^{2}$ ≥ 0.1) and within 500 kb of the lead SNPs were clumped. This process was repeated iteratively for the next top index SNPs, excluding those already clumped, until no index SNPs remained. Subsequently, the lead SNP was selected for each LD block, ensuring that all lead SNPs were both independent and significant. We then identified lead SNP pairs (one SNP from each trait) that were located within 500 Kb of each other. For these paired SNPs, we calculated their LD. If $r^{2}$ ≥ 0.8, they were classified as shared loci^(11)^.

CPASSOC uses summary-level GWAS statistics from single-SNP-trait associations to detect variants associated with at least one trait, providing two statistics, S_Hom_ and S_Het_. S_Het_ was chosen due to its ability to account for heterogeneity effects^(12)^. PLEIO, on the other hand, identifies and interprets pleiotropic loci in joint analyses of complex traits by utilizing GWAS summary statistics.

For CPASSOC and PLEIO, we used the PLINK clumping function to target independent pleiotropic SNPs: a significance threshold for index SNPs of P < 5×10⁻⁸ (--clump-p1 5e-8), a secondary significance threshold for clumped SNPs of P < 1×10⁻⁵ (--clump-p2 1e-5), a minimum $r^{2}$ threshold of 0.2 (--clump-r2 0.2) and a maximum distance of 500 Kb (--clump-kb 500^)(10)^. Significant index SNPs were recognized with *P*_CPASSOC/PLEIO_ < 5×10⁻⁸ and *P*_single-trait_ < 1×10⁻^3^. The identified significant index SNPs were further classified into four groups: (1) “known” shared SNPs, which were significant in both traits (*P_CAC_* < 5×10⁻⁸ and *P_eBMD_* < 6.6×10⁻^9^); (2) “single-trait-driven” shared SNPs, significant in only one trait (either *P_CAC_* < 5×10⁻⁸ or *P_eBMD_* < 6.6×10⁻^9^); (3) “LD-tagged” shared SNPs, not significant in either trait (*P_CAC_* ≥ 5×10⁻⁸ and *P_eBMD_* ≥ 6.6×10⁻^9^), but in LD ($r^{2}$ ≥ 0.2) or located within 500 kb of significant SNPs reported by single-trait GWAS; (4) “novel” shared SNPs, which were of most interest, defined as those that were neither significant in both traits nor in LD with reported significant SNPs.

To determine whether the shared variants are causally responsible for two GWAS signals, we performed a colocalization analysis using **COLOC**, a novel Bayesian statistical procedure that yields five posterior probabilities; if the posterior probability for H4 (PPH4) of the shared locus exceeded 0.70, this locus was colocalized^(13)^.

We used Ensembl Variant Effect Predictor (VEP)^(14)^ and 3DSNP^(15)^ for gene annotation of the index SNPs.

**MAGMA analysis (multi-SNP-level analysis)**

Gene-based analysis offers a promising alternative to the single-SNP analysis in GWAS, particularly for complex traits with polygenic backgrounds predominantly influenced by numerous variants with minor effects. To perform a multi-SNP-level analysis, we applied Multi-marker Analysis of GenoMic Annotation (**MAGMA**), which annotates SNPs at the gene-wide level^(16)^. MAGMA uses a multiple linear principal components regression to incorporate LD between genetic markers and quantifies the association of all markers within a gene and a phenotype. In this analysis, we applied the SNP-wise mean model to compute gene-level associations. Gene location files (NCBI37.3) and reference data (1000 Genomes European panel) were downloaded from the MAGMA website. The SNP *P*-values extracted from GWAS summary data were used as input for gene analysis. Significant genes with *P* < 1×10⁻^3^ from both traits were merged to identify shared genes, which were subsequently classified according to whether they had been previously reported.

**Transcriptome-wide association study (gene expression level)**

To identify associations between transcriptome gene expression in specific tissues and traits, we conducted a transcriptome-wide association study (**TWAS**) using FUSION based on expression weights from 49 GTEx (Genotype-Tissue Expression, version 8) tissues^(17)^. TWAS analyzes the relationship between gene expression predicted by genetic variants and complex traits, combining predicted gene expression levels with GWAS summary statistics to identify genes with cis-regulated expression linked to complex traits^(17)^. A Bonferroni correction was applied within each tissue to account for multiple comparisons. Significantly associated genes (*P*_Bonferroni_ < 0.05) from both traits were merged to identify shared gene expressions.

**Sensitivity Analysis Using DXA-Derived BMD GWAS**

Given the marked difference in sample sizes between the CAC GWAS (~27,000 individuals) and the eBMD GWAS (~427,000 individuals), cross-trait meta-analyses may be biased by an inflated type I error rate, potentially leading to eBMD-driven loci. Furthermore, heel ultrasound–derived eBMD correlates only modestly with axial BMD and may not fully reflect the genetic architecture of clinically relevant skeletal sites, such as the femoral neck and lumbar spine. To address these limitations and improve clinical interpretability, we conducted a sensitivity analysis using DXA-derived BMD GWAS.

The FN BMD GWAS (N = 49,988) and LS BMD GWAS (N = 44,731) were conducted in individuals of European ancestry, with bone mineral density measured using dual-energy X-ray absorptiometry (DXA)^(18)^. Summary statistics for both FN and LS BMD were subjected to the same multi-layered pleiotropy analysis as the primary study, including CPASSOC, PLEIO, MAGMA, and TWAS. Significance thresholds were set as nominal *P* < 0.05. This approach allowed us to validate whether the key pleiotropic loci identified in the primary eBMD-based analysis.

**Pathway enrichment analyses**

To further analyze the functions of significant shared genes identified through cross-trait analysis, MAGMA, and TWAS, we performed pathway enrichment analyses, including protein-protein interaction (**PPI**) network, Gene Ontology (**GO**)^(19,20)^ and Kyoto Encyclopedia of Genes and Genomes (**KEGG**)^(21)^ analysis. For PPI network construction, all significant shared genes were entered into the STRING database (version 12.0, https://string-db.org/) to identify protein interactions^(22)^. Cytoscape software (version 3.7.1) was used to determine hub genes and visualize the network^(23)^. The hub genes were identified by assessing the “degree” of each node, which means the number of direct connections or interactions, with higher degree values indicating greater connectedness and influence within the network. GO analysis categorizes genes across three biological domains—biological process, cellular component, and molecular function—allowing for a detailed annotation of gene roles in cellular and physiological contexts. KEGG analysis, in contrast, provides insights into molecular functions within broader biological systems, linking genes to specific pathways involved in complex disease processes. We conducted GO and KEGG analyses using the R language clusterProfiler package^(24)^, with *P* < 0.05 as statistical significance.

**Reference:**

1. Kavousi M, Bos MM, Barnes HJ, et al., Multi-ancestry genome-wide study identifies effector genes and druggable pathways for coronary artery calcification. Nat Genet. Oct 2023;55(10):1651.

2. Agatston AS, Janowitz WR, Hildner FJ, et al., Quantification of coronary artery calcium using ultrafast computed tomography. J Am Coll Cardiol. Mar 15 1990;15(4):827.

3. Morris JA, Kemp JP, Youlten SE, et al., An atlas of genetic influences on osteoporosis in humans and mice. Nat Genet. Feb 2019;51(2):258.

4. Bulik-Sullivan B, Finucane HK, Anttila V, et al., An atlas of genetic correlations across human diseases and traits. Nat Genet. Nov 2015;47(11):1236.

5. Zhang Y, Lu Q, Ye Y, et al., SUPERGNOVA: local genetic correlation analysis reveals heterogeneous etiologic sharing of complex traits. Genome Biol. Sep 7 2021;22(1):262.

6. Yin L, Zhang H, Tang Z, et al., rMVP: A Memory-efficient, Visualization-enhanced, and Parallel-accelerated Tool for Genome-wide Association Study. Genomics Proteomics Bioinformatics. Aug 2021;19(4):619.

7. Turley P, Walters RK, Maghzian O, et al., Multi-trait analysis of genome-wide association summary statistics using MTAG. Nat Genet. Feb 2018;50(2):229.

8. Zhu X, Feng T, Tayo BO, et al., Meta-analysis of correlated traits via summary statistics from GWASs with an application in hypertension. Am J Hum Genet. Jan 8 2015;96(1):21.

9. Lee CH, Shi H, Pasaniuc B, et al., PLEIO: a method to map and interpret pleiotropic loci with GWAS summary statistics. Am J Hum Genet. Jan 7 2021;108(1):36.

10. Purcell S, Neale B, Todd-Brown K, et al., PLINK: a tool set for whole-genome association and population-based linkage analyses. Am J Hum Genet. Sep 2007;81(3):559.

11. Rahmioglu N, Mortlock S, Ghiasi M, et al., The genetic basis of endometriosis and comorbidity with other pain and inflammatory conditions. Nat Genet. Mar 2023;55(3):423.

12. Li X, Zhu X, Cross-Phenotype Association Analysis Using Summary Statistics from GWAS. Methods Mol Biol. 2017;1666:455.

13. Giambartolomei C, Vukcevic D, Schadt EE, et al., Bayesian test for colocalisation between pairs of genetic association studies using summary statistics. PLoS Genet. May 2014;10(5):e1004383.

14. Cunningham F, Allen JE, Allen J, et al., Ensembl 2022. Nucleic Acids Res. Jan 7 2022;50(D1):D988.

15. Quan C, Ping J, Lu H, et al., 3DSNP 2.0: update and expansion of the noncoding genomic variant annotation database. Nucleic Acids Res. Jan 7 2022;50(D1):D950.

16. de Leeuw CA, Mooij JM, Heskes T, et al., MAGMA: generalized gene-set analysis of GWAS data. PLoS Comput Biol. Apr 2015;11(4):e1004219.

17. Gusev A, Ko A, Shi H, et al., Integrative approaches for large-scale transcriptome-wide association studies. Nat Genet. Mar 2016;48(3):245.

18. Zheng HF, Forgetta V, Hsu YH, et al., Whole‐genome sequencing identifies EN1 as a determinant of bone density and fracture. Nature. 2015;526(7571):112.

19. Ashburner M, Ball CA, Blake JA, et al., Gene ontology: tool for the unification of biology. The Gene Ontology Consortium. Nat Genet. May 2000;25(1):25.

20. Gene Ontology C, Aleksander SA, Balhoff J, et al., The Gene Ontology knowledgebase in 2023. Genetics. May 4 2023;224(1).

21. Kanehisa M, Furumichi M, Sato Y, et al., KEGG for taxonomy-based analysis of pathways and genomes. Nucleic Acids Res. Jan 6 2023;51(D1):D587.

22. Szklarczyk D, Kirsch R, Koutrouli M, et al., The STRING database in 2023: protein-protein association networks and functional enrichment analyses for any sequenced genome of interest. Nucleic Acids Res. Jan 6 2023;51(D1):D638.

23. Shannon P, Markiel A, Ozier O, et al., Cytoscape: A Software Environment for Integrated Models of Biomolecular Interaction Networks. Genome Research. 2003;13(11):2498.

24. Yu G, Wang LG, Han Y, et al., clusterProfiler: an R package for comparing biological themes among gene clusters. OMICS. May 2012;16(5):284.
